# Unmasking the Hidden Threat: A Case Report of the Anesthetic Management of a Catecholamine-Secreting Tumor in Pregnancy

**DOI:** 10.7759/cureus.72611

**Published:** 2024-10-29

**Authors:** Daniela Sepúlveda, Rita Gonçalves Cardoso, Susana Santos Rodrigues, Neuza de Sousa, Marta Pereira

**Affiliations:** 1 Anesthesiology, Unidade Local de Saúde do Alto Ave, Guimarães, PRT

**Keywords:** catecholamine-secreting tumor, gestational hypertension, paraganglioma, perioperative management, pheochromocytoma

## Abstract

Catecholamine-secreting tumors (CSTs) are rare but serious conditions, particularly in pregnancy, as they significantly increase the risk of maternal and fetal morbidity and mortality. Diagnosing CSTs in pregnant women with hypertension is challenging due to overlapping symptoms, with more common conditions like preeclampsia. In this report, we present the case of a 24-year-old hypertensive primigravida, diagnosed with a CST at 27 weeks of gestation following previous hypertensive episodes. Her management involved pretreatment with alpha- and beta-blockers to control hypertension, followed by an elective cesarean section at 32 weeks. The combined neuraxial anesthesia approach ensured stable hemodynamics during surgery, and a healthy newborn was delivered. Three months after the delivery, tumor resection was performed, confirming the diagnosis of multicentric sympathetic paraganglioma. This case highlights the intricate challenges of managing CSTs during pregnancy. The survival of both the mother and the fetus relies heavily on timely diagnosis, appropriate medical interventions, optimal perioperative management, and the precise timing of both delivery and surgical procedures. These decisions should be made on an individual basis, and close collaboration between obstetricians, endocrinologists, anesthesiologists, and surgeons is essential. Given the rarity of CSTs in pregnancy, this case highlights the importance of maintaining a high index of suspicion in hypertensive pregnant patients and the need for individualized care strategies.

## Introduction

Catecholamine-secreting tumors (CSTs) are rare neuroendocrine tumors originating either from the chromaffin cells of the adrenal medulla, known as pheochromocytomas, or from the extra-adrenal autonomic ganglia, referred to as paragangliomas (PGLs) [1].

Although CSTs are uncommon during pregnancy, they pose significant risks, potentially resulting in life-threatening consequences for both the mother and the fetus [2]. Due to the high amount of placental catechol-O-methyltransferase and monoamine oxidase, the fetus is not exposed to maternal circulating catecholamine levels [1,3]. However, placental exposure to maternal hypertensive episodes can lead to placental vasoconstriction with repercussions on uteroplacental perfusion [1].

The diagnostic challenge is multifactorial, stemming from the tumor’s rarity, its variable clinical presentations, and limitations imposed by pregnancy on certain imaging modalities and radioisotope studies. Additionally, uterine growth during pregnancy may be responsible for triggering tumor activity, which until then was silent [1,4].

The literature on this subject is predominantly anecdotal, with no established consensus or guidelines for optimal management, making the CST during pregnancy a complex therapeutic challenge.

A case of a CST in pregnancy managed at our institution is presented, discussing the medical pretreatment, the timing of adrenalectomy, delivery planning, and perioperative management.

## Case presentation

A 24-year-old White pregnant woman, gravida 1, para 0, with a history of hypertension, morbid obesity (BMI = 42 kg/m^2^), and gestational diabetes was admitted to the hospital during her 22nd gestational week due to poorly controlled hypertension.

At the time of her hypertension diagnosis, at the age of 19, the patient was being followed at another hospital for evaluation of potential secondary hypertension. During this period, her blood pressure was controlled with azilsartan 40 mg and chlorthalidone 25 mg. The patient presents a family history of premature cardiovascular disease, with her father having experienced a stroke at the age of 41. Several causes of secondary hypertension, including primary hyperaldosteronism and renovascular disease, had been ruled out, and an abdominal computed tomography (CT) scan revealed a 20-mm adenoma in the right adrenal gland. However, the diagnostic process remained inconclusive due to the patient’s missed follow-up appointments.

The patient became pregnant and was treated for her chronic hypertension of unknown cause with 250 mg of methyldopa three times a day and 30 mg of nifedipine twice a day. Regarding her gestational diabetes, she was treated with metformin 500 mg twice daily, achieving good glycemic control. At 22 weeks of gestation, during a routine antenatal appointment, she was found to be hypertensive (173/119 mmHg) and tachycardic (111 bpm), and the 24-hour urine collection showed proteinuria of 306 mg/24 hours (normal range, <300 mg/24 hours). Due to the suspicion of superimposed preeclampsia on chronic hypertension, she was admitted to the obstetrics ward. The endocrinology team was consulted, and the investigation for secondary hypertension was resumed. The patient was discharged home with ongoing diagnostic tests, and her medication was adjusted, with the morning dose of nifedipine increased to 60 mg.

**Figure 1:**
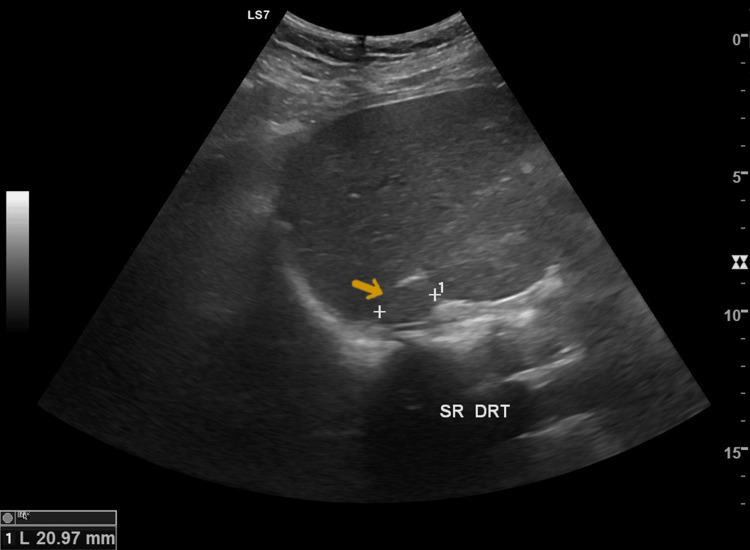
Abdominal ultrasonography revealing a 21-mm nodule in the right adrenal gland (arrow)

At 27 weeks of gestation, the suspicion of a catecholamine-producing tumor was confirmed by elevated plasma normetanephrine levels of 1155 pg/mL (upper limit of normal, 196 pg/mL) and 24-hour urinary normetanephrine levels of 3679 µg/24 hours (upper limit of normal, 444 µg/24 hours). The patient was readmitted for optimization of her therapy. The abdominal ultrasonography revealed a 21-mm nodule in the right adrenal gland (Figure 1). However, magnetic resonance imaging (MRI) could not be performed due to the patient’s inability to cooperate due to claustrophobia. During this hospital stay, her previous antihypertensive medications were discontinued, and her treatment was adjusted and titrated to a dose of 16 mg of doxazosin twice daily and 20 mg of propranolol three times daily. This new regimen, in combination with a low-sodium diet, allowed for acceptable blood pressure control (systolic pressure, 121-143 mmHg; diastolic pressure, 79-92 mmHg).

Throughout the hospitalization, there was a progressive increase in proteinuria levels in the 24-hour urine samples (at 28 weeks, 1265 mg/24 hours; at 31 weeks, 11805 mg/24 hours; normal range, <300 mg/24 hours). Additionally, the patient developed peripheral edema and new-onset renal dysfunction, evidenced by an increase in serum creatinine to 1.29 mg/dL (baseline, 0.85 mg/dL). Given her clinical deterioration, a multidisciplinary meeting involving anesthesiology, obstetrics, neonatology, and endocrinology was held, and a cesarean section was scheduled for 32 weeks of gestation. Corticosteroid therapy was initiated to achieve fetal lung maturity.

**Figure 2:**
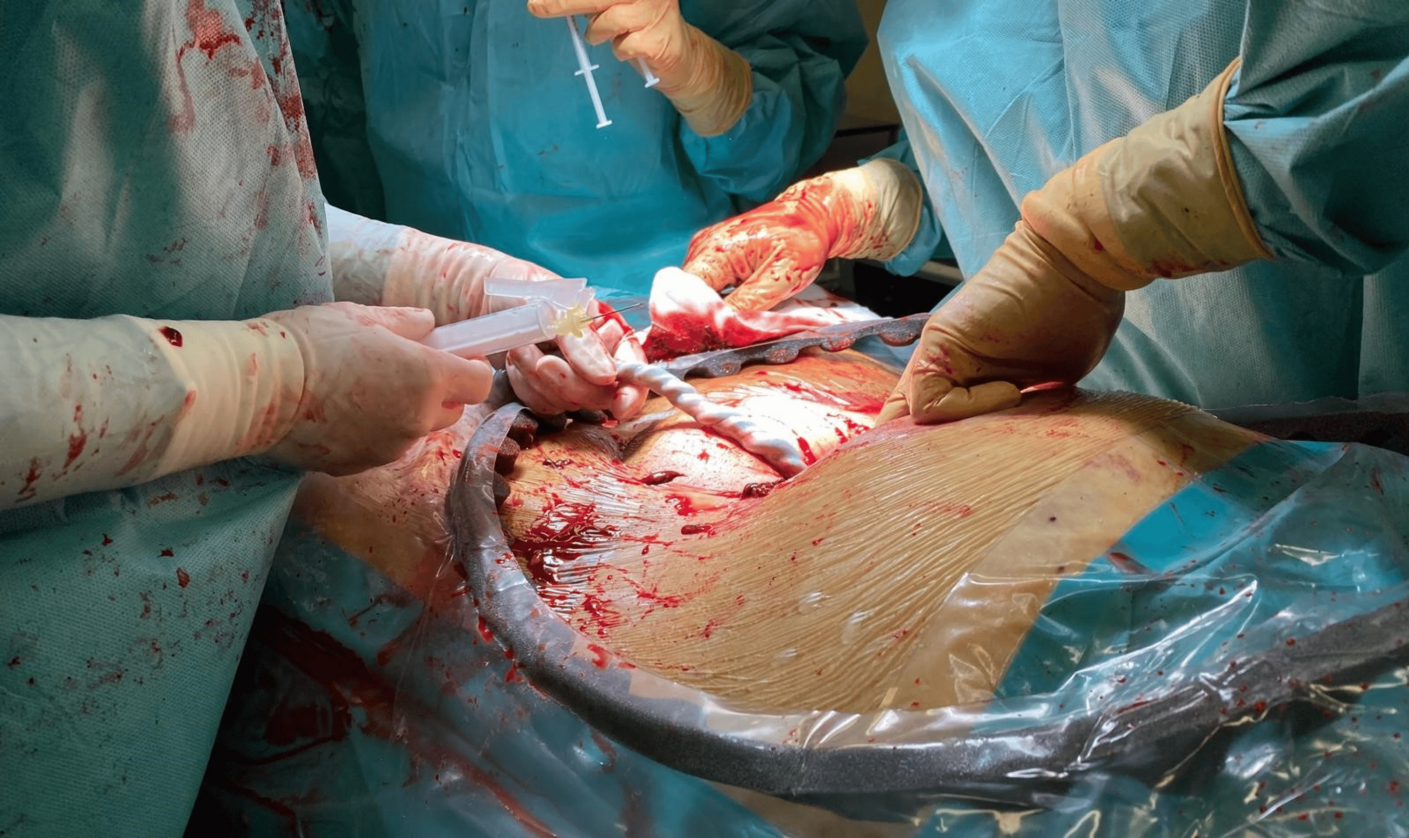
Intraoperative phase of the cesarean section

Regarding the anesthetic approach for the cesarean section, a combined neuraxial technique was chosen. The patient was thoroughly and timely informed about all the procedures that would be performed. In the operating room, a left radial arterial line and a right internal jugular central venous line were inserted under local anesthesia with ultrasound guidance. She was calm and cooperative, thus avoiding the need for premedication. An epidural catheter was placed in the left lateral decubitus position at the L3-L4 intervertebral space, using an 18 G Tuohy needle with loss-of-resistance to air technique. Subsequently, a subarachnoid block was performed with a 27 G Quincke needle, with the administration of 2 mL of 0.5% hyperbaric bupivacaine and 2 mcg of sufentanil, achieving a satisfactory level of anesthetic blockade for cesarean section (Figure 2). During the procedure, 4 g of magnesium sulfate was administered and her blood pressure remained stable. A 1910-g healthy girl with an Apgar score of 7/8/9 was born. The newborn was admitted to the neonatal intensive care unit due to prematurity and maternal doxazosin therapy. The admission was uneventful.

In the first few days of the postpartum period, the patient remained under observation in a level II care unit. Magnesium sulfate infusion was continued for 24 hours post-delivery. Despite effective pain management with epidural analgesia, the patient experienced episodes of elevated blood pressure (175/103 mmHg), which were successfully managed with boluses of labetalol. 

**Figure 3:**
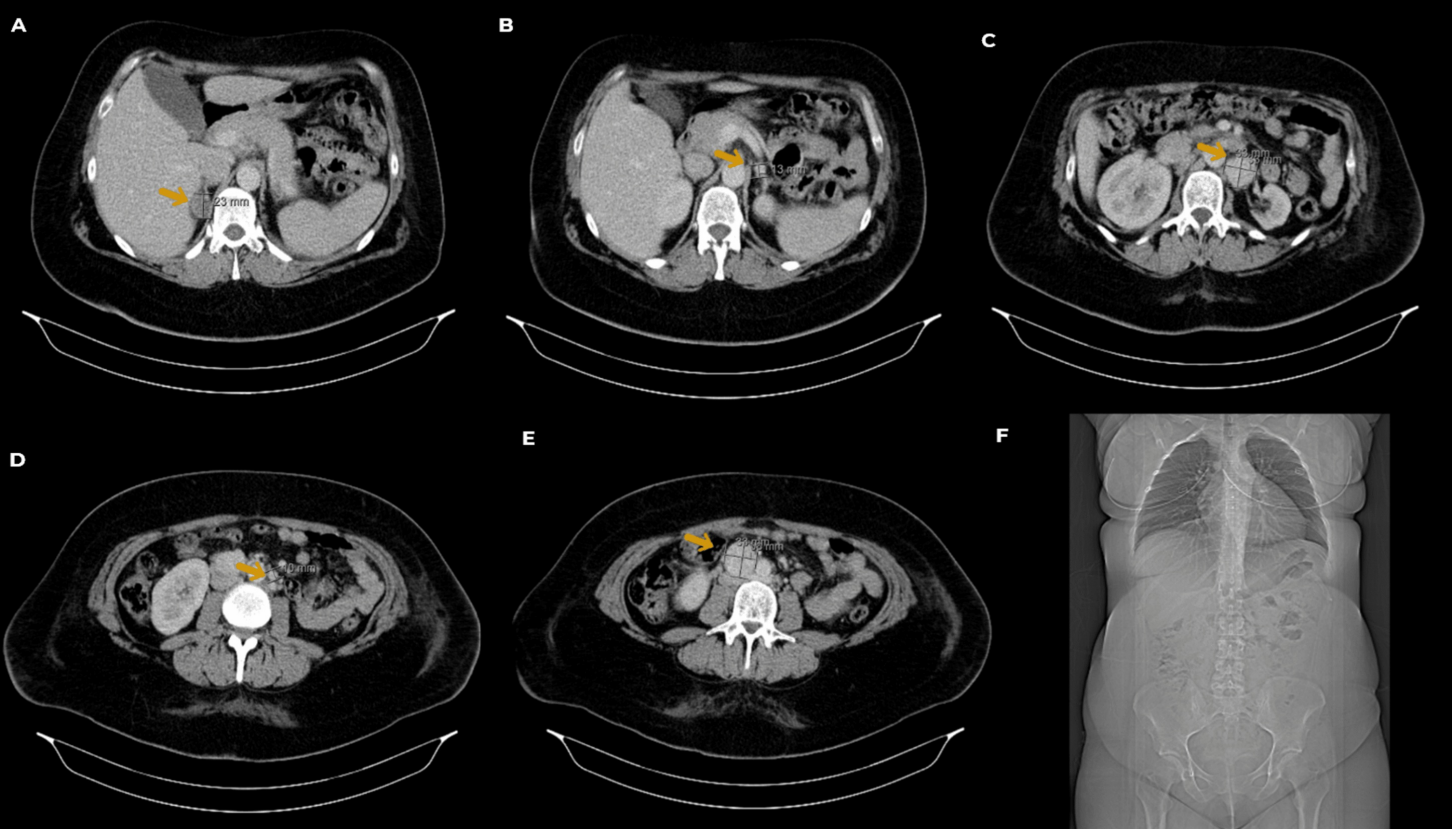
Contrast-enhanced abdominopelvic CT scan performed postpartum The scan revealed the following findings (arrow): (A) a 23-mm mildly enhancing solid nodule in the right adrenal gland, suggestive of an adenoma; (B) a 13-mm hyperenhancing solid nodule adjacent to the left adrenal gland; (C) and (D) two lymph nodes in the left para-aortic region, measuring 33 mm and 10 mm; (E) a 36-mm lymph node in the inter-aortocaval region; and (F) CT scout view providing an overview of the anatomical positioning prior to detailed imaging.

Postpartum, a contrast-enhanced abdominopelvic CT scan revealed a 23-mm mildly enhancing solid nodule in the right adrenal gland, suggestive of an adenoma, as well as a 15-mm hyperenhancing solid nodule adjacent to the left adrenal gland, raising suspicion of pheochromocytoma/PGL. Additionally, three hyperenhancing lymph nodes were identified: two in the left para-aortic region, measuring 33 mm and 10 mm, and one measuring 36 mm in the inter-aortocaval region, consistent with metastatic involvement (Figure 3). These findings were subsequently confirmed by a 68Ga-DOTANOC PET/CT scan, which showed overexpression of somatostatin receptors in the left adrenal gland and the three previously described lymph nodes. No functionally suspicious lesions with receptor overexpression were observed in other sites, including the right adrenal gland.

A genetic study, including a disease-exome-based next-generation sequencing panel targeting 17 genes (CDKN1B, EGLN1, FH, GDNF, KIF1B, MAX, MEN1, NF1, RET, SDHA, SDHAF2, SDHB, SDHC, SDHD, TMEM127, VHL, and PRKAR1A), and an MLPA analysis of exon 1 were conducted. Both tests yielded negative results. No further genetic testing was currently available.

Metformin was discontinued after the delivery, and good glycemic control was maintained. Regarding blood pressure management, the patient continued her ongoing alpha- and beta-antagonist medication. Three months postpartum, she underwent an open left adrenalectomy with dissection of the described lymph nodes under general anesthesia. She remained hemodynamically stable throughout the surgery. The excised nodules, including the one located near the left adrenal gland, were histologically characterized as a multicentric, extra-adrenal, sympathetic PGL.

During hospitalization, the patient’s hypertension persisted, with systolic blood pressure ranging from 121 to 160 mmHg and diastolic pressure from 80 to 105 mmHg. Fourteen days after the surgical removal, the patient was discharged from the hospital on a medication regimen that included enalapril 20 mg and lercanidipine 20 mg daily, as well as carvedilol 6.25 mg twice a day, with a plan for gradual tapering. Instructions were provided for monitoring heart rate and blood pressure at home. Additionally, a urinary metanephrine test and a follow-up appointment were scheduled for one month after the surgery.

At the follow-up appointment, urinary normetanephrine levels remained elevated at 1900 µg/24 hours (upper limit of normal at 444 µg/24 hours). New imaging studies were requested, including 68Ga-DOTANOC and 18F-FDG PET/CT scans, which were performed two months post-surgery and revealed three different hypercaptant retroperitoneal lesions. The size of the previously reported right adrenal nodule remained the same and showed no uptake. In light of these findings, indicating a diagnosis of metastatic PGL, the patient resumed doxazosin at a dose of 8 mg twice daily and reduced the doses of enalapril and lercanidipine by half.

**Figure 4:**
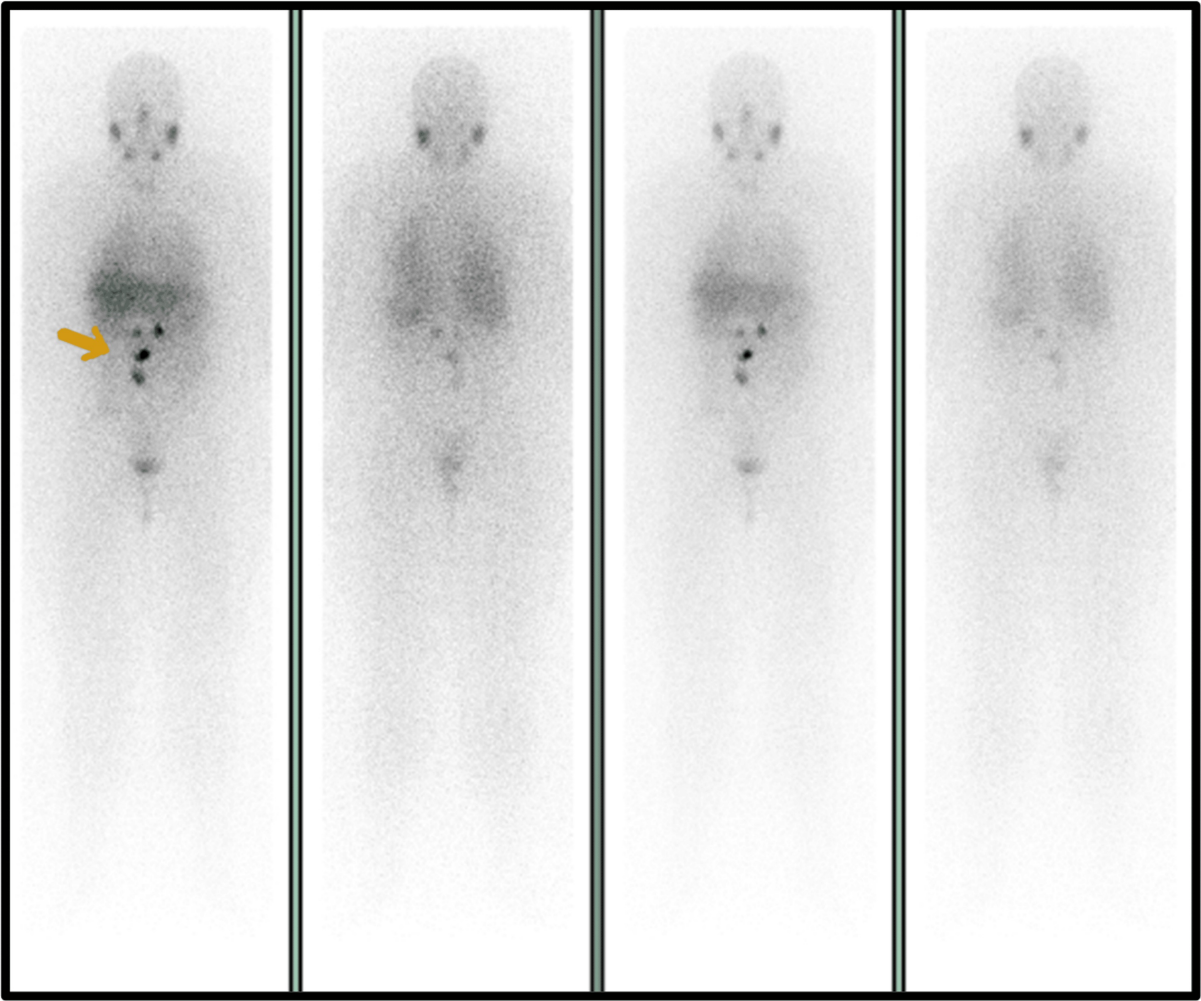
Postoperative scintigraphy showing abnormal ¹²³I-MIBG uptake Postoperative scintigraphy revealed four foci of abnormal ¹²³I-MIBG uptake: two in the epigastric/mesogastric transition and two in the mesogastric region.

To establish a treatment strategy, a ^123^I-MIBG scintigraphy was performed, which revealed four lymph node lesions exhibiting abnormal uptake in the abdominal region (Figure 4). Given the diagnosis of a metastatic PGL, with prior surgery deemed non-curative and the presence of additional detectable lesions on the ^123^I-MIBG scan, the therapeutic approach proposed for the patient was 131I-MIBG therapy.

## Discussion

CSTs during pregnancy are an extremely rare occurrence. However, despite their rarity, promptly diagnosing CSTs in hypertensive pregnant women is of paramount importance, as untreated cases can lead to maternal and fetal mortality rates of up to 40-50% [1,2,4-7]. Hypertension, however, is a common complication in pregnancy, making it challenging to distinguish a CST from more frequent causes of pregnancy-related hypertension, such as gestational hypertension and preeclampsia. Certain clinical features, such as severe hypertension before 20 weeks of gestation and/or paroxysmal hypertension interspersed with periods of hypotension, should heighten suspicion for CSTs and warrant further diagnostic evaluation [1,5].

In this case, although the investigation for secondary hypertension had not been completed, there was already a suspicion of a CST before the pregnancy. The suspicion was only confirmed at 27 weeks of gestation, when elevated plasma and urinary concentrations of normetanephrine were detected, along with an ultrasound revealing nodules in the adrenal glands. When elevated levels of urinary or plasma metanephrines or normetanephrines are detected, additional imaging is required for diagnosis. For imaging diagnosis, ultrasound and MRI are the only safe options during pregnancy, with MRI being the first-line choice as it does not involve ionizing radiation, similar to ultrasound, while providing excellent visualization of the abdomen, pelvis, neck, and mediastinum [1,5]. However, due to the patient’s morbid obesity and claustrophobia, she was unable to tolerate the MRI scan, and a more detailed evaluation through CT was only possible postpartum.

The therapeutic approach for a patient with CSTs is based on two main pillars: pretreatment with alpha-adrenergic receptor blockade and the surgical removal of the tumor [8].

Pretreatment with α-adrenergic receptor blockade should be initiated as early as the diagnosis is made and at least 10 to 14 days prior to surgery, in order to reduce the blood pressure, paroxysms, and perioperative complications [8,9]. CST management during pregnancy is no exception but has the particular requirement of ensuring the preservation of uteroplacental circulation. The alpha-adrenergic receptor blocker dose titration requires a balance between managing hypertension and ensuring that uteroplacental circulation is not compromised [6] 

The choice of a specific alpha-blocker must be carefully considered in the treatment of CSTs during pregnancy. Phenoxybenzamine is a nonselective and irreversible alpha-adrenergic antagonist with a half-life of 24 hours, and its clinical effects may last up to a week after discontinuation [5,10]. The presynaptic alpha-2 blockade caused by phenoxybenzamine can increase norepinephrine release from presynaptic nerves, leading to reflex tachycardia in the mother [1]. Another concern with phenoxybenzamine is that it crosses the placenta and accumulates in the fetus, which is a disadvantage since catecholamines do not pass widely through the placenta due to catecholamine uptake and degradation by receptors and enzymes present in placental cells [5]. This accumulation can potentially lead to neonatal respiratory depression and hypotension.

Doxazosin is a selective, competitive alpha-1 adrenergic blocker with a plasma half-life of 20 hours. Although doxazosin can cross the human placental barrier, neonatal hypotension and respiratory depression have not been reported. The advantages of doxazosin over phenoxybenzamine include a lower incidence of reflex tachycardia and reduced postoperative hypotension [1,10,11].

Although doxazosin was chosen for our patient due to its potential advantages, there was still a need to add beta-blockade to counteract catecholamine-induced tachyarrhythmias and the reflex tachycardias induced by α-adrenergic receptor blockade. When no tachycardia is present, calcium channel blockers are an alternative if hypertension is not successfully stabilized with α-adrenergic receptor blockers [8].

Labetalol and methyldopa are no longer recommended in pregnant patients with hypertension due to pheochromocytoma; however, our patient was on methyldopa until the diagnosis of a CST, at 27 weeks, at which point methyldopa was discontinued and doxazosin was initiated [1]. Another critical measure is the restoration of intravascular volume in a way that reduces the risk of large fluctuations in blood pressure during surgery and the risk of prolonged postoperative hypotension while supporting uteroplacental circulation [1,6,8].

Surgery is the definitive treatment for CSTs; however, during pregnancy, determining the optimal timing is critical. Ideally, given the diagnostic suspicion before pregnancy, treatment should have been undertaken before conception. In this case, due to missed follow-up appointments and a 27-week pregnancy, we were confronted with two options: performing tumor removal during pregnancy or postponing surgery until after the delivery.

According to the literature, when the diagnosis is made within the first 24 weeks of pregnancy, surgical resection is generally recommended in the second trimester, after organogenesis is complete. If diagnosed in the third trimester and medical management is effective, surgery is typically delayed until after delivery [6,11]. Our patient’s diagnosis was confirmed around 27 weeks of pregnancy, and preoperative optimization was necessary. Since she was already in the third trimester, there was no option but to postpone tumor resection until after the delivery, as the enlarged uterus reduces access to the tumor, increasing the surgical risk in the third trimester [1,5,6].

The optimal route of delivery for patients with CSTs remains a subject of debate, as there is insufficient evidence to establish definitive recommendations. Some studies have reported successful vaginal deliveries under epidural analgesia in women with pheochromocytoma [10]. Pregnant patients in whom vaginal delivery may be considered include those with small tumors, located outside the abdominopelvic cavity, as well as multiparous women where a faster labor is anticipated [12,13].

In the literature, cesarean section has traditionally been favored in such cases. This method offers clear advantages, particularly the ability to perform the surgery in a controlled setting, allowing for continuous hemodynamic monitoring and the capacity to swiftly address any hemodynamic instability that may arise. However, despite these advantages, cesarean delivery is not without its risks. There is considerable potential for hemorrhage due to the uterine incision, along with the heightened risk of catecholamine release, which may be triggered by surgical manipulation of the peritoneum or the tumor, potentially intensifying hypertensive crises and leading to further complications [14].

The decision regarding the route of delivery should be highly individualized, taking into account several key factors, such as the patient’s obstetric history, the current maternal and fetal conditions, the patient’s clinical response to preoperative treatment, and her personal preferences. Additionally, the timing of delivery is crucial and may be influenced by the patient’s overall clinical status, fetal maturity, and the availability of a multidisciplinary team experienced in managing such high-risk cases [1,5].

In the case of our patient, the clinical picture worsened, and considering the need for a highly skilled team to be readily available, an elective cesarean section was scheduled at 32 weeks of gestation. This decision was based not only on the maternal condition but also on optimizing fetal outcomes, ensuring the least risk for both the mother and the child. In circumstances such as these, the balance between maternal safety and fetal viability must always be carefully weighed, and the delivery plan should be adaptable to the evolving clinical scenario.

Performing a cesarean section in a patient with a CST presents a considerable challenge for the anesthesiologist, as uterine manipulation can trigger catecholamine release, leading to intraoperative hypertension, which in turn increases both maternal and perinatal morbidity and mortality. Regarding the anesthetic technique, epidural, spinal, general, and combined anesthetic techniques have been described to be safe in pregnant women with pheochromocytoma [14].

General anesthesia may be a valid option as it provides a more controlled environment for managing potential surgical complications such as hemorrhage. However, general anesthesia carries a significant risk of catecholamine surges during airway manipulation, which heightens the likelihood of dangerous hypertensive episodes [14,15]. Additionally, airway management in morbidly obese patients can be technically challenging, raising the risk of difficult intubation, particularly in a patient considered to have a full stomach. If general anesthesia is chosen, all induction agents are generally considered safe, except ketamine, which can stimulate the sympathetic nervous system [14,15]. Furthermore, it is recommended to avoid non-depolarizing muscle relaxants with vagolytic or histamine-releasing properties. Maintenance of anesthesia can be achieved with volatile agents such as isoflurane or sevoflurane, while halothane should be avoided due to its sensitization of the myocardium to circulating catecholamines, and desflurane is discouraged as it can provoke catecholamine release when administered rapidly [14-17].

Regional anesthesia, such as epidural or spinal, is often preferred due to its ability to provide effective pain control while minimizing the systemic effects associated with general anesthesia, especially avoiding the hypertensive crises that can be triggered by intubation and extubation. Nonetheless, regional anesthesia is not without risks. Sympathetic stimulation resulting from patient anxiety may still occur, and hypotension is a potential complication. Most studies favor continuous epidural anesthesia over spinal block due to the lower risk of profound hypotension [15]. However, in this case, given the abdominal location of the tumor, a combined neuraxial technique was preferred. This approach allowed for a denser anesthetic block, providing optimal surgical conditions on a morbidly obese patient, while also enabling the titration of the anesthetic level and offering the benefit of postoperative analgesia through the use of an epidural catheter.

In addition to the medications previously mentioned in the context of general anesthesia, other drugs can trigger the release of catecholamines and precipitate a hypertensive crisis and should therefore be avoided, such as metoclopramide, steroids, and sympathomimetics [1,16,17].

In the perioperative period, the patient was stable but had an episode of rising blood pressure to 150/100 mmHg, probably explained by the surgical manipulation during the cesarean section. Taking advantage of the administration of 4 g of magnesium sulfate as preeclampsia prophylaxis, the arterial blood pressure normalized to 139/85 mmHg. The patient maintained hemodynamic stability throughout the procedure. Magnesium sulfate is commonly employed in the management of preeclampsia. It acts as a calcium channel antagonist, promotes direct vasodilation, and additionally acts by blocking peripheral catecholamine receptors, which helps decrease catecholamine release from CSTs [1,2,5,12].

In the case of acute hypertension, emergency medication must be prepared for administration. Phentolamine, a competitive antagonist of both α1 and α2 receptors, can be useful in treating severe hypertension induced by catecholamines due to its rapid onset of action [1,5,12]. However, it has been associated with significant maternal hypotension and maternal-fetal mortality, thus should be used with caution and reserved for emergency situations [12]. Other alternatives include short-acting calcium channel blockers such as nicardipine or magnesium sulfate, which can be administered safely [1,2,5,12]. If tachycardia is present, rapid-acting beta-blockers like labetalol and esmolol may be given, but only after achieving adequate α-blockade [1,12]. Direct vasodilators such as hydralazine, nitroglycerin, and sodium nitroprusside can also be effective in treating hypertensive crises; however, hydralazine is generally discouraged due to its potential to precipitate acute coronary syndrome in patients with pheochromocytoma [12]. The use of nitroprusside during pregnancy should be avoided due to the theoretical risk of fetal cyanide toxicity [1,5].

Support must be provided to the pregnant patient throughout the entire peripartum period, with a particular focus on psychological assistance, as emotional instability can trigger sympathetic activation. Keeping our patient informed about the decisions made and thoroughly explaining all procedures helped to alleviate her anxiety, ultimately eliminating the need for premedication at the time of the cesarean section. This proactive approach proved beneficial for both the mother and the baby.

In young women of reproductive age, PGL is more commonly linked to an underlying germline pathogenic variant [13]. In this case, despite a strong clinical suspicion, genetic testing did not reveal any germline variants. Thus, a hereditary predisposition syndrome could not be confirmed, though it cannot be entirely excluded.

It is recommended to measure plasma or urine levels of metanephrines during postoperative follow-up to diagnose persistent disease. Initial biochemical tests should be conducted once the patient has sufficiently recovered from surgery, typically 2 to 4 weeks after the procedure, to confirm successful tumor removal. Once remission criteria are met, it is recommended to undergo annual biochemical testing for life to track any possible recurrence or metastatic disease [8]. In this case, the postpartum surgery was not curative, as indicated by the initial positive biochemical assessment and confirmed by imaging studies, which ultimately revealed a metastatic PGL.

Generally, the clinical criteria for evaluating the suitability of radio-targeted therapy with 131I-MIBG encompass metastatic disease affecting multiple sites, progressive disease, or symptomatic manifestations. Treatment eligibility is largely determined through imaging assessments, particularly with 123I-MIBG scans [18]. In this case, a 123I-MIBG scan was conducted, and the substantial uptake observed led to a recommendation for 131I-MIBG therapy for the patient.

The patient’s surgery was not curative, but alternative treatment options are available, and her prognosis will depend on her response to the proposed therapies. If there is no response to MIBG treatment, other alternatives such as systemic chemotherapy or targeted therapies may be considered. Furthermore, chronic treatment with an alpha-blocker may be necessary if prolonged remission is not achieved [18].

## Conclusions

Managing CSTs during pregnancy presents a unique and complex challenge. Early and accurate diagnosis and effective preoperative optimization using alpha- and beta-adrenergic blockades and strategic planning of both timing and method of delivery are critical in mitigating associated risks. This case report highlights the effectiveness of a combined neuraxial anesthetic approach in achieving a stable and safe elective cesarean section for a patient with a CST. Surgical intervention to address the primary tumor was performed postpartum, with timing guided by the gestational age at diagnosis. Optimized management of such rare pathologies requires a carefully individualized approach, coordinated by a multidisciplinary team of specialists, to ensure optimal maternal and fetal outcomes.

## References

[1] Prete A, Paragliola RM, Salvatori R, Corsello SM (2016). Management of catecholamine-secreting tumors in pregnancy: a review. Endocr Pract.

[2] Merve K, Emre K, Hakan D (2019). Pregnancy and pheochromocytoma. Med J Clin Trials Case Stud.

[3] Dahia PL, Hayashida CY, Strunz C, Abelin N, Toledo SP (1994). Low cord blood levels of catecholamine from a newborn of a pheochromocytoma patient. Eur J Endocrinol.

[4] Oliva R, Angelos P, Kaplan E, Bakris G (2010). Pheochromocytoma in pregnancy: a case series and review. Hypertension.

[5] van der Weerd K, van Noord C, Loeve M (2017). Endocrinology in pregnancy: Pheochromocytoma in pregnancy: case series and review of literature. Eur J Endocrinol.

[6] Lenders JW (2012). Pheochromocytoma and pregnancy: a deceptive connection. Eur J Endocrinol.

[7] Ghalandarpoor-Attar SN, Ghalandarpoor-Attar SM, Borna S, Ghotbizadeh F (2018). A rare presentation of pheochromocytoma in pregnancy: a case report. J Med Case Rep.

[8] (2023). Correction to "Pheochromocytoma and paraganglioma: an Endocrine Society Clinical Practice Guideline". J Clin Endocrinol Metab.

[9] Witteles RM, Kaplan EL, Roizen MF (2000). Safe and cost-effective preoperative preparation of patients with pheochromocytoma. Anesth Analg.

[10] Wing LA, Conaglen JV, Meyer-Rochow GY, Elston MS (2015). Paraganglioma in pregnancy: a case series and review of the literature. J Clin Endocrinol Metab.

[11] Mannelli M, Bemporad D (2002). Diagnosis and management of pheochromocytoma during pregnancy. J Endocrinol Invest.

[12] Gruber LM, Young WF Jr, Bancos I (2021). Pheochromocytoma and paraganglioma in pregnancy: a new era. Curr Cardiol Rep.

[13] Bancos I, Atkinson E, Eng C, Young WF Jr, Neumann HP (2021). Maternal and fetal outcomes in phaeochromocytoma and pregnancy: a multicentre retrospective cohort study and systematic review of literature. Lancet Diabetes Endocrinol.

[14] Cammarano WB, Gray AT, Rosen MA, Lim KH (1997). Anesthesia for combined cesarean section and extra-adrenal pheochromocytoma resection: a case report and literature review. Int J Obstet Anesth.

[15] Stonham J, Wakefield C (1983). Phaeochromocytoma in pregnancy. Caesarean section under epidural analgesia. Anaesthesia.

[16] Rosas AL, Kasperlik-Zaluska AA, Papierska L, Bass BL, Pacak K, Eisenhofer G (2008). Pheochromocytoma crisis induced by glucocorticoids: a report of four cases and review of the literature. Eur J Endocrinol.

[17] Kamoun M, Mnif MF, Charfi N (2014). Adrenal diseases during pregnancy: pathophysiology, diagnosis and management strategies. Am J Med Sci.

[18] Sukrithan V, Perez K, Pandit-Taskar N, Jimenez C (2024). Management of metastatic pheochromocytomas and paragangliomas: when and what. Curr Probl Cancer.

